# Analysis of androgen receptor and anti-Müllerian hormone pathways in human granulosa cells under luteinizing hormone treatment

**DOI:** 10.1186/1477-7827-11-11

**Published:** 2013-02-21

**Authors:** Kuo-Chung Lan, Shiuh Young Chang, Fu-Jen Huang, Hsin-Jung Lin, Ching-Yuang Lin, Ko-En Huang, Hong-Yo Kang

**Affiliations:** 1 Department of Obstetrics and Gynecology, Kaohsiung Chang Gung Memorial Hospital and Chang Gung University College of Medicine, Kaohsiung, Taiwan; 2 Graduate Institute of Clinical Medical Sciences, Chang Gung University, Kaohsiung, Taiwan; 3 Center for Menopause and Reproductive Medicine Research, Kaohsiung Chang Gung Memorial Hospital and Chang Gung University College of Medicine, Kaohsiung, Taiwan; 4 Sheng An Hospital, 177 Mei-Sui East 2nd Road, Kaohsiung, Taiwan; 5 College of Medicine, China Medical University, Clinical Immunology Center, China Medical University Hospital, Taichung, Taiwan

**Keywords:** Controlled ovarian stimulation, Recombinant luteinizing hormone, Androgen receptor, SOX9, Anti-Müllerian hormone

## Abstract

**Background:**

The objective of this study was to determine the gene expression profiles of the androgen/androgen receptor (AR) and anti-Müllerian hormone (AMH)/ Sry-related high-mobility group box 9 (SOX9) pathways in granulosa-luteal cells from patients undergoing standard *in vitro* fertilization (IVF) with or without recombinant luteinizing hormone (rLH) therapy.

**Methods:**

Levels of reproductive hormones in the pre-ovulatory follicular fluid and the expression levels of LHR (luteinizing hormone receptor), AR, SOX9, AMH, AR-associated protein 54(ARA54)and ARA70 were determined in granulosa-luteal cells by real-time reverse-transcription PCR. The effects of androgen and rLH treatments on AR and AMH expression levels were also tested *in vitro* using HO23 cells.

**Results:**

We collected 35 an 70 granulosa cell samples from patients cycled with and without rLH supplementation, respectively. The clinical outcomes were similar in patients who received rLH therapy and those who did not, though the pre-ovulatory follicular fluid levels of androstenedione, testosterone, and estradiol were significantly higher and progesterone was lower in the rLH supplementation group. Moreover, granulosa-luteal cell mRNA levels of LHR, AR, AMH, and SOX9 were significantly higher in the rLH supplementation group relative to the group that did not receive rLH supplementation. In addition, we observed significant correlations between LHR and AR mRNA expression and among AR, AMH, and SOX9 mRNA expression in granulosa-luteal cells from patients undergoing standard IVF treatment.

**Conclusions:**

Increased expression of LHR, AR, AMH, and SOX9 is characteristic of granulosa-luteal cells from IVF/ intracytoplasmic sperm injection (ICSI) patients receiving rLH supplementation.

## Background

Controlled ovarian hyperstimulation (COH) is one of the pivotal steps in assisted reproductive technology (ART). The role of endogenous luteinizing hormone (LH) levels and the effect of LH on follicular maturation and pregnancy outcome during ovarian stimulation have attracted a great deal of attention since the early years of *in vitro* fertilization (IVF) [1,2]. With the availability of recombinant human LH (rLH), clinicians now have the opportunity to administer two gonadotropins independently. Thus, exogenous rLH administration can be calibrated independently of recombinant follicle-stimulating hormone (rFSH).

The use of rLH for COH with respect to indications, timing, and dosage has not been fully elucidated [3,4]. A growing body of evidence seems to indicate a beneficial effect of co-treatment with rFSH and rLH, in particular for patients suffering pregnancy loss, poor responders [5-8] and patients of advanced age who undergo ART [6,9-13]. However, because of the relatively small sample sizes, many of these trials were underpowered for evaluating clinical pregnancy as a primary outcome and were thus inconclusive as to the benefit of rLH therapy*.* To better establish the role of LH in IVF, additional basic research and randomized, focused clinical trials are needed [9,14,15].

Ovarian androgens are produced by thecal cells and exert their action in a paracrine fashion on granulosa cells. Androgen action is mediated by the androgen receptor (AR), which, like other members of the steroid receptor superfamily, interacts directly with target genes to regulate transcription [16]. Several coactivator proteins have been shown to bind steroid receptors and enhance their interaction with basal transcription factors, thereby amplifying the transcriptional activation potential of the receptor. Among these coactivators are the AR-associated protein 54 (ARA54) and ARA70 [17]. We previously proposed the possibility of a transition in androgen action from an enhancer of follicular differentiation (through the AR) to a substrate for estrogen synthesis (through aromatase) at the time of oocyte retrieval [17].

Sry-related high-mobility group box (SOX) proteins make up a large family of transcription factors that share a homologous high-mobility group (HMG) DNA-binding domain and are key regulators of many developmental and tissue-specific processes. In the developing gonad, SOX9 plays a critical role in male sex determination by stimulating the expression of anti-Müllerian hormone (AMH) [18]. Recent clinical evidence showed that serum AMH levels might be a sensitive predictive parameter of ovarian status [19]. Evidence indicates a negative role for AMH of pre-granulosa/granulosa cell origin in this key event and subsequent progression to the antral stage [20]. Whether intrafollicular AMH is involved in the autocrine/paracrine regulation of granulosa and theca cell function in pre-ovulatory antral follicles remains unclear. Notably, the poor responders who benefit from co-treatment with rFSH and rLH [3,5-8] usually also exhibit extremely low serum AMH levels [19]. The “two-cell-two-gonadotropin” theory posits that ovarian theca cells stimulated by LH produce androgens, which are then converted by granulosa cells into estrogens under FSH stimulation [21]. However, the action of LH on follicular development is unlikely to be limited to providing an androgen substrate for aromatization. In fact, LH also direct stimulates and modulates folliculogenesis [22]. However, the efficacy of rLH in the follicular fluid microenvironment and on luteinized granulosa cells has been less thoroughly assessed, and studies focusing on the interaction between LH, androgen/AR and AMH/SOX9 in granulosa cells are limited [23-27]. Our aim in this study was to examine whether rLH therapy affects the gene expression profiles of the androgen/AR or AMH/SOX9 endocrine and paracrine hormonal signaling pathways in human luteinized granulosa cells.

We measured the expression levels of the luteinizing hormone receptor (LHR), AR, AR coactivators, SOX9, and AMH in granulosa cells collected from IVF/ICSI patients on the gonadotropin-releasing hormone agonist (GnRHa) protocol for ovarian stimulation, with or without rLH co-treatment with rFSH. In addition, we treated the human granulosa cell line HO-23 with rLH or androgen in vitro to investigate whether the gene expression profiles of the AR and AMH pathways were regulated by both LH and androgen.

## Methods

### COH Protocol

**Figure 1:**
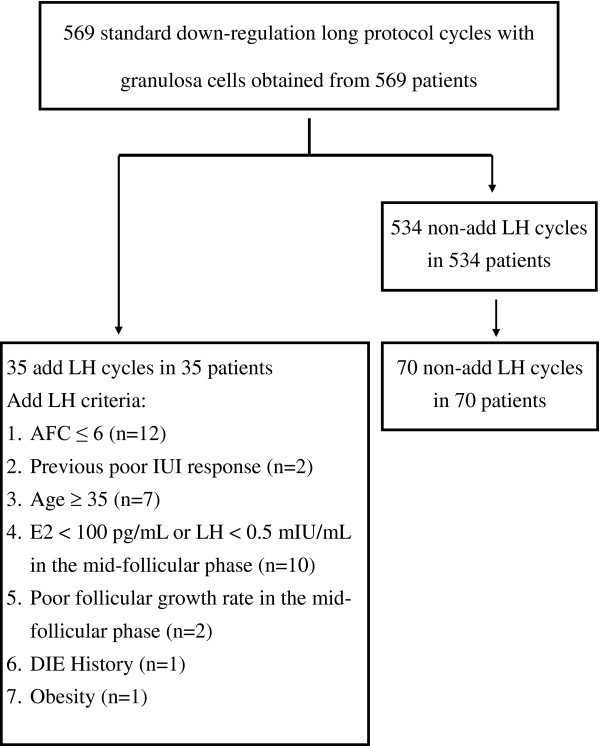
Flow diagram of 539 patients who attended the case-control study.

539 Patients undergoing their first ART treatment with a long COH protocol were enrolled in the study. This study was approved by the Ethics Committee of Chang Gung Memorial Hospital. Approval from the institutional review board was obtained for the analysis of this series (CGMH94-360). The study included patients who underwent IVF/intracytoplasmic sperm injection in our institution between March 1, 2004 and September 30, 2006. Within this study period, 35 patients received rLH supplementation cycles (12 patients with antral follicle count (AFC) <6; 2 patients with previous intrauterine insemination (IUI) poor response history; 7 patients with age > 35 years old; 10 patients with mid-follicular serum estradiol <100 pg/mL or LH < 0.5mIU/mL; 2 patients with poor follicular growth rate in the mid-follicular phase; 1 patient with deep infiltrating endometriosis history and 1 patient due to obesity). To match appropriate candidates to compare outcome efficiency, a 2:1 individual matching case-control design [28] was used to recruit 70 patients who did not receive rLH supplementation cycles but matched the demographic characteristics of the 35 patients receiving rLH therapy (Figure1). At that time, it has been suggested that recombinant LH can be used in a group of unselected IVF patients [29]. All patients provided informed consent for their participation in the molecular investigation. The COH procedure was followed by the standard down-regulation protocol, as published previously [30]. Briefly, GnRHa (Lupron; Abbott Australasia PTY, Ltd., Kurnell, New South Wales, Australia) was administered from the mid-luteal phase, and rFSH (Gonal-F; Serono Laboratories, Aubonne, Switzerland) was administered after pituitary desensitization was achieved. Patients received one of the following fixed starting doses (age < 35: 225 IU/day; age≥35: 300 IU/day) of rFSH (Gonal-F, 75 IU/ampoule, Serono Laboratories, Aubonne, Switzerland) after pituitary suppression with GnRHa. Gonadotropin was administered daily for 5 days, after which the dose was individualized according to ovarian follicular growth, as in our previous reports [30]. Monitoring of transvaginal ultrasound and serum estradiol (E2) was performed every 2 to 3 days starting on the 5^th^ day of stimulation. After 6 days of rFSH stimulation, patients who had ever received co-treatment with 75 IU of rLH (Luveris; Merck Serono SA, Aubonne Branch, Switzerland) were submitted to the rLH supplementation group. rFSH, with or without rLH, was continuously administered until 2 of the dominant follicles reached 18 mm, and then the patients received 250 μg of hCG (Ovidrel; Merck Serono, Inc., West Orange, NJ, USA).

### Collection of primary human granulosa cells

Thirty-four to 36 hours after hCG administration, follicular fluid (FF) and luteinized granulosa cells were collected by means of transvaginal ultrasound-guided oocyte retrieval. A double-lumen needle was used for aspiration because the puncture needle used to obtain a sample of uncontaminated luteinized granulosa cells was not withdrawn before entering the next follicle. Only the first aspirate of pre-ovulatory follicles without blood contamination from one ovary was used for this study. After follicular aspiration, the cumulus-oocyte complexes were separated for IVF, and the luteinized granulosa cells were flushed with IVF medium to disperse the cells. The cells were then immediately transported to the laboratory. The follicular fluid was stored under the same conditions as serum. The FF samples were analyzed for testosterone, estradiol, progesterone, and androstenedione. The dispersed cells were transferred to a 15-mL centrifuge tube containing 4 mL of Histopaque 1077 (Sigma Chemical Co., St. Louis, MO, USA). Human luteinized granulosa cells were separated from red blood cells by centrifugation at 600 × g for 10 minutes. Granulosa cells formed a thin layer between the Histopaque and the medium [31] and were collected as previously reported [17].

### Cell lines and culture conditions

The HO23 human immortalized luteinized granulosa cell line, provided by Dr. Abraham Amsterdam, Weizmann Institute of Science, Rehovot, Israel [32], was used for *in vitro* analyses. HO23 cells were established by triple transfection of SV40 DNA, the Ha-*ras* oncogene, and a temperature-sensitive (ts) mutant of the p53 tumor suppressor gene (p53val135) in primary granulosa cells obtained from *IVF* patients. The cells were maintained at 37°C and 5% CO_2_ in Dulbecco’s minimal essential medium (DMEM)/Ham’s F12 (1:1), supplemented with 5% fetal calf serum and antibiotics (100 IU/mL penicillin and 100 mg/mL streptomycin). To test the effect of rLH or androgen on AR and AMH expression, cells (5 × 10^5^) were seeded on 100-mm culture dishes and incubated for 24 hours. The media were then removed, and the cells were reincubated in medium supplemented with charcoal-dextran-treated serum and then treated with different concentrations of DHT or rLH for 24 hours.

### RNA extraction

Total RNA was extracted from granulosa cells (5 × 10^5^) using TRIzol reagent (Gibco-BRL, Grand Island, NY, USA). Briefly, 1 mL of TRIzol was added to the granulosa cells. The mixture was pipetted and allowed to sit for 5 minutes at room temperature. Chloroform (0.2 mL) was added, mixed, and allowed to incubate at room temperature for 3 minutes. The mixture was then processed by centrifugation at 12,000 × g for 15 minutes, and the supernatant was transferred to a fresh tube. Isopropanol (0.5 mL) was added, mixed, and incubated for 10 minutes at room temperature. The solution was then processed by centrifugation at 12,000 × g for 10 minutes, and the RNA was purified.. The pellet was washed once with 70% ethanol, resuspended in H_2_O, and stored at -80°C. The concentrations of the RNA samples were determined by measuring the absorbance at 260 nm in a spectrophotometer (DU® 640B, Beckman Coulter, USA).

### cDNA synthesis

Two micrograms of total RNA from the sample preparation were reverse-transcribed in 25 μL as follows: 0.1 μg of random hexamer primers (Amersham Pharmacia Biotech, Inc., Buckinghamshire, UK) were denatured for 10 minutes at 70°C in a Gradient Cycler (DNA Engine, Watertown, MA, USA), and then the reverse transcription reaction was performed at 42°C for 1 hour by adding 5× reverse transcriptase buffer (500 mM of each dNTP, 3 mM MgCl_2_, 75 mM KCl, and 50 mM Tris-HCl [pH 8.3]), 1 mM dNTP (Promega, Madison, WI, USA), 10 IU RNase inhibitor (Promega), and 100 U MMLV reverse transcriptase (Promega). The reverse transcriptase was inactivated by heating at 95°C for 5 minutes and cooling at 4°C for 5 minutes.

### Quantitative RT-PCR

Specific PCR amplification products were detected using an ABI PRISM 7700 sequence detector system (Perkin-Elmer Applied Biosystems, Los Angeles, CA, USA) and the SYBR green PCR master mix kit (Applied Biosystems, Foster City, USA), according to the manufacturer’s protocol. The forward and reverse primer sequences for AR, AR co-factors, SOX9, AMH, LHR and ß-actin and 18S are listed in Table 1. Duplicate experiments were performed for each set of experimental conditions, and we retested any sample with a > 1% coefficient of variation for the Ct value. Quantitative values are obtained from the threshold cycle (*Ct*) number at which the increase in signal associated with an exponential growth of PCR product starts to be detected (using 7500 Fast System SDS software (Applied Biosystems)), according to the manufacturer’s manual. The precise amount of total RNA added to each reaction (based on absorbance) and its quality (*i.e.*, lack of extensive degradation) are both difficult to assess. We quantified ß-actin or 18S gene transcripts as an endogenous RNA control, and normalized each sample with respect to its ß-actin or 18S content. Final results, express as *N*-fold differences in target gene expression relative to the β-actin gene termed “*N* target,” are determined as follows: *N* target=2^△Ct sample^where the △Ct values of the sample were determined by subtracting the average Ct value of the target gene from the average Ct value of the ß-actin or 18S gene in each sample.

**Table 1:** Oligonucleotide primer sequences for LHR, AR, AMH, SOX9, ARA54, ARA70 and β-actin used in real-time quantitative RT-PCR

**Gene**	**Oligo-sequence(5**^**′**^ → **3**^**′**^)
LHR	F: TCA.ATT.CTT.GTG.CCA.ATC.CA
	R: CCA.TTT.TTG.CAG.TTG.GAG.GT
AR	F: TCA.CCG.CAC.CTG.ATG.TGT.G
	R: ACA.TGG.TCC.CTG.GCA.GTC.TC
AMH	F: AGC.TGT.GGG.CAC.CAG.TGG.
	R: GCT.CTT.GTG.GGC.TGC.CTG.
SOX 9	F: GCA.AAG.GAG.ATG.AAA.TCT.GTT.CTG
	R: AAG.GTT.AAC.TGC.TGG.TGT.TCT.GAG.A
ARA54	F: TCT.GCC.TCC.ACT.TGT.GCT.GA
	R: GCC.ACG.GTG.TTC.TTC.CCA.TA
ARA70	F: TGG.AGC.TTG.CTA.TTG.GTG.GAG
	R: CAG.GTG.ACG.GCT.TAT.GCA.ACT
ß-actin 18S	F: TCA.CCC.ACA.CTG.TGC.CCA.TCT.ACG.A
	R: CAG.CGG.AAC.CGC.TCA.TTG.CCA.ATG.G
	F:GTA.ACC.CGT.TGA.ACC.CCA.TT
	R:CCA.TCC.AAT.CGG.TAG.TAG.CG

### Statistical analysis

Data analyses were performed with SPSS 10.0 software (Statistical Package for Social Sciences, Inc., Chicago, IL, USA). Continuous data were summarized as the mean ± standard deviation (SD). For the purpose of this analysis, correlations in gene expression were examined. A multiple regression analysis with the stepwise forward procedure (multivariate analysis) was used to identify independent factors and to test for interactions between the covariates. The clinical outcome comparison used the Mann-Whitney rank-sum test for the comparison of means and the Fisher’s exact test for proportions. All *P* values were two-sided, and *P* < 0.05 was considered statistically significant.

## Results

The study population comprised two groups of patients. One group (35 patients) received rLH supplementation cycles during the IVF procedure, while the control group (70 patients) did not. The two groups were comparable in terms of age, BMI, mean duration of infertility, indication for treatment, days of FSH treatment, endometrial thickness, estradiol and progesterone levels on the day of hCG administration, and the number of oocytes and mature oocytes retrieved (Table 2).

**Table 2:** Comparison of patients treated with Add-rLH or Non-Add-rLH IVF down-regulation regimens

**Transfer**	**Add-rLH cycles**	**Non-Add-rLH cycles**	**P**
*No. patients*	35	70	
*Age of female partners (yrs)*	33.4 +/- 4.1	33.2 +/- 4.2	ns
*Body mass index*	21.5 +/- 3.1	21.6 +/- 3.1	ns
*Infertility*			
*Primary (%)*	68.6 (24/35)	55.7 (39/70)	ns
*Secondary (%)*	31.4 (11/35)	44.3 (31/70)	ns
*Reason for infertility (No. of patients)*			
*Tubal factor*	11	23	ns
*Male factor*	17	32	ns
*Unexplained*	3	2	ns
*Combined factor*	4	13	ns
*Duration of infertility (years)*	4.0 +/- 2.4	4.0 +/- 3.0	ns
*Days of FSH treatment*	8.6 +/- 1.8	9.1 +/- 1.7	ns
*FSH (IU)*	2062.5 +/- 975.0	2385.0 +/- 855.0	ns
*LH (IU)*	3525.0 +/- 225.0		
*Endometrial thickness on day of hCG stimulation (cm)*	1.4 +/- 0.3	1.3 +/- 0.3	ns
*LH (mIU/mL) on day of hCG stimulation*	2.6 +/- 1.6	1.9 +/- 1.2	0.021
*Estradiol (pg/mL) on day of hCG stimulation*	1681.9 +/- 1021.7	1851.8 +/- 1166.2	ns
*Progesterone (ng/mL) on day of hCG stimulation*	1.2 +/- 0.4	1.1 +/- 0.4	ns
*No. mature & near-mature oocytes retrieved*	5.9 +/- 4.2	6.5 +/- 3.3	ns
*No. mature oocytes retrieved*	3.7 +/- 2.7	3.9 +/- 2.1	ns
*Normal fertilization rate*	77.8 +/- 33.1%	79.4 +/- 23.3%	ns
*Mean No. embryos transferred*	2.1 +/- 0.6	2.3 +/- 0.6	ns
*Clinical pregnancy rate per transfer*	34.2% (12/35)	32.8% (23/70)	ns

*Note*: Values are mean ± SD or proportion.

We found that the fertilization rate, mean number of embryos transferred, and clinical pregnancy rate per transfer were similar between the two groups. The serum LH level on the day of hCG administration was the only parameter to show a statistically significant difference between the two groups (2.6 +/- 1.6 mIU/mL vs. 1.9 +/- 1.2 mIU/mL, *P =0.021*; Table 2). However, the concentrations of androstenedione, testosterone, estradiol and progesterone in the pre-ovulatory dominant follicular fluid were significantly different between groups. Follicular fluid levels of androstenedione (76.4+/- 43.2 vs. 54.1+/- 42.6), testosterone (10.1+/- 3.5 vs. 9.4 +/- 6.0), and estradiol (336619.8 +/- 366528.9 vs.195014.7 +/- 257784.7) were higher, and progesterone was lower (10337.9 +/- 5138.1 vs. 11974.4 +/- 20806.6) in the rLH supplementation groups (Table 3).

**Table 3:** Comparison of follicular fluid concentrations at oocyte retrieval in patients treated with Add-LH or Non-Add rLH IVF down-regulation regimens

**Transfer**	**Add-rLH cycles**	**Non-Add rLH cycles**	**P**
No. patients	35	70	
Androstenedione (ng/mL)	76.4+/- 43.2	54.1+/- 42.6	0.009
Testosterone (ng/mL)	10.1+/- 3.5	9.4 +/- 6.0	0.021
Estradiol (pg/mL)	336619.8 +/- 366528.9	195014.7+/- 257784.7	0.034
Progesterone (ng/mL)	10337.9 +/- 5138.1	11974.4+/-20806.6	0.007

*Note*: Measurements were taken on the day of oocyte retrieval. Values are mean ± SD.

**Figure 2:**
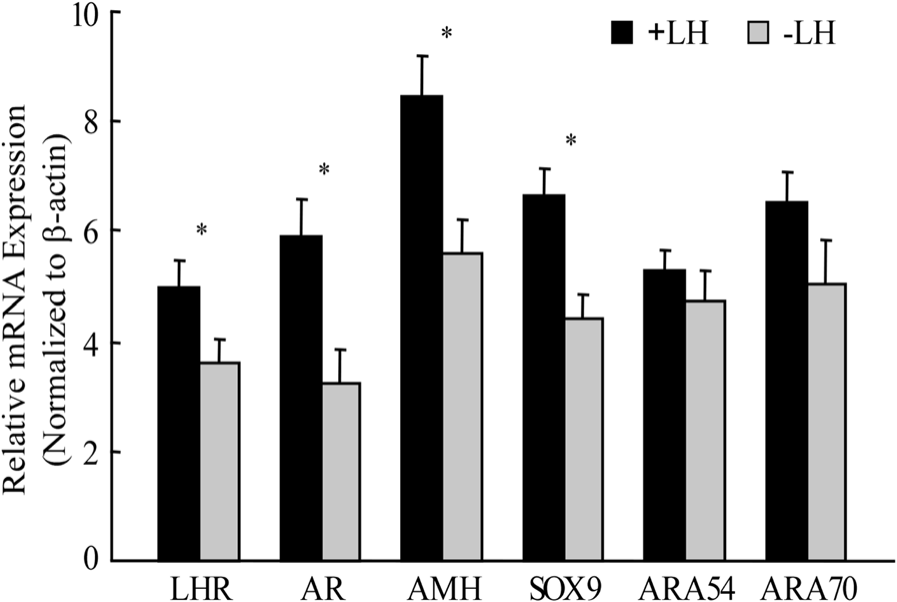
**Quantitative analysis of LHR, AR, AMH, SOX9, ARA54 and ARA70 mRNA levels in granulosa-luteal cells collected at oocyte retrieval.** Each bar represents the mean ± SD. Concomitant detection of β-actin mRNA in the real-time RT-PCR reaction served as a reference for relative quantification. * *P* < 0.05.

**Figure 3:**
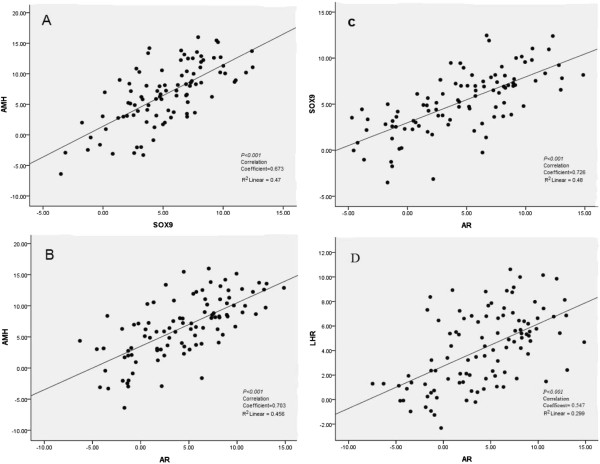
**Linear regression revealed positive correlations in gene expression patterns.** (**A**) AMH and SOX9 expression; (**B**) AMH and AR expression; (**C**) SOX9 and AR expression; and (**D**) LHR and AR expression.

**Figure 4:**
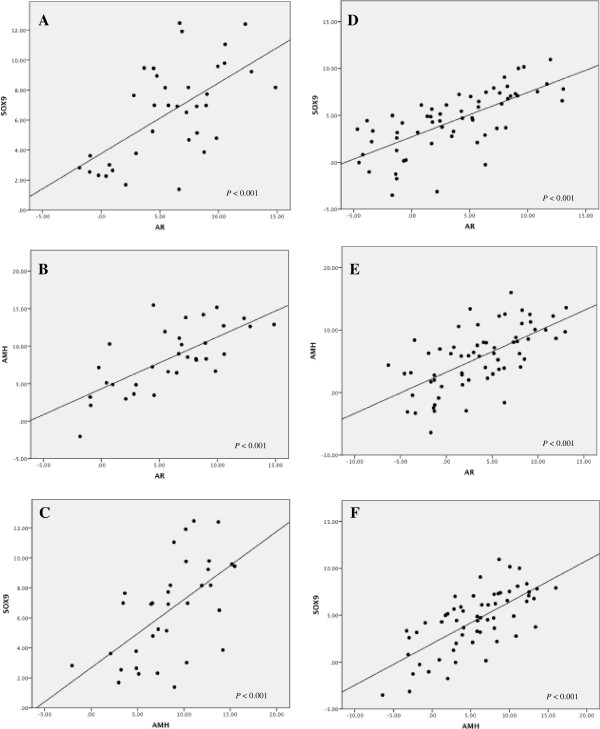
**Linear regression revealed positive correlations in gene expression patterns in LH (A-C) and non-add LH (D-F) groups.** (**A**) SOX9 and AR expression; (**B**) AMH and AR expression; (**C**) AMH and SOX9expression (**D**) SOX9 and AR expression; (**E**) AMH and AR expression; (**F**) AMH and SOX9expression.

We next examined whether the expression levels of potential genes involved in the androgen and AMH pathways were influenced by rLH supplementation during COH. The mRNA levels of the LH receptor (LHR), AR, SOX9, AMH, ARA54 and ARA70 were quantified by real-time RT-PCR in luteinized granulosa cell samples obtained from women who received (35 patients) or did not receive rLH supplementation (70 patients). The results are summarized in Figure 2 and Additional file 1: Figure S1. Of note, the expression of LHR, AR, SOX9, and AMH was significantly higher in granulosa cells obtained from women who received rLH supplementation. AR co-regulators, i.e., ARA70 and ARA54, were expressed at similar levels between groups. Linear regression revealed that AMH, SOX9 and AR expression levels in granulosa cells were positively correlated (Figure 3, Figure 4 and Table 4). ARA54 and ARA70 expression levels were significantly correlated with SOX9 and AMH, respectively (Table 4). There was also a positive correlation between LHR and AR expression (Figure 3 and Table 4).

**Table 4:** Linear regression analysis of LHR, AR, AMH, SOX9, ARA54 and ARA70 mRNA expression in granulosa-luteal cells collected at oocyte retrieval

**Comparison variable**	**B**	**95.0% Confidence interval for B**	**P**
		**LHR**	
**AR**	0.352	0.236; 0.468	<0.001
**AMH**			ns
**SOX9**			ns
**ARA70**			ns
**ARA54**			ns
	**AR**		
**AMH**	0.326	0.160; 0.492	<0.001
**SOX9**	0.514	0.262; 0.766	<0.001
**LHR**	0.348	0.129; 0.568	0.002
**ARA70**			ns
**ARA54**			ns
	**SOX9**		
**AR**	0.299	0.168; 0.429	<0.001
**AMH**	0.227	0.105; 0.348	<0.001
**LHR**			ns
**ARA70**			ns
**ARA54**	0.218	0.094; 0.341	0.001
	**AMH**		
**AR**	0.370	0.153; 0.587	0.001
**SOX9**	0.517	0.227; 0.807	0.001
**LHR**			ns
**ARA70**	0.181	0.032; 0.330	0.018
**ARA54**			ns
	**ARA70**		
**AR**			ns
**SOX9**			ns
**LHR**	0.388	0.062; 0.715	0.020
**AMH**	0.467	0.104; 0.619	<0.001
**ARA54**			ns
	**ARA54**		
**AR**			ns
**SOX9**	0.483	0.280; 0.686	<0.001
**LHR**			ns
**AMH**			ns
**ARA70**			ns

**Figure 5:**
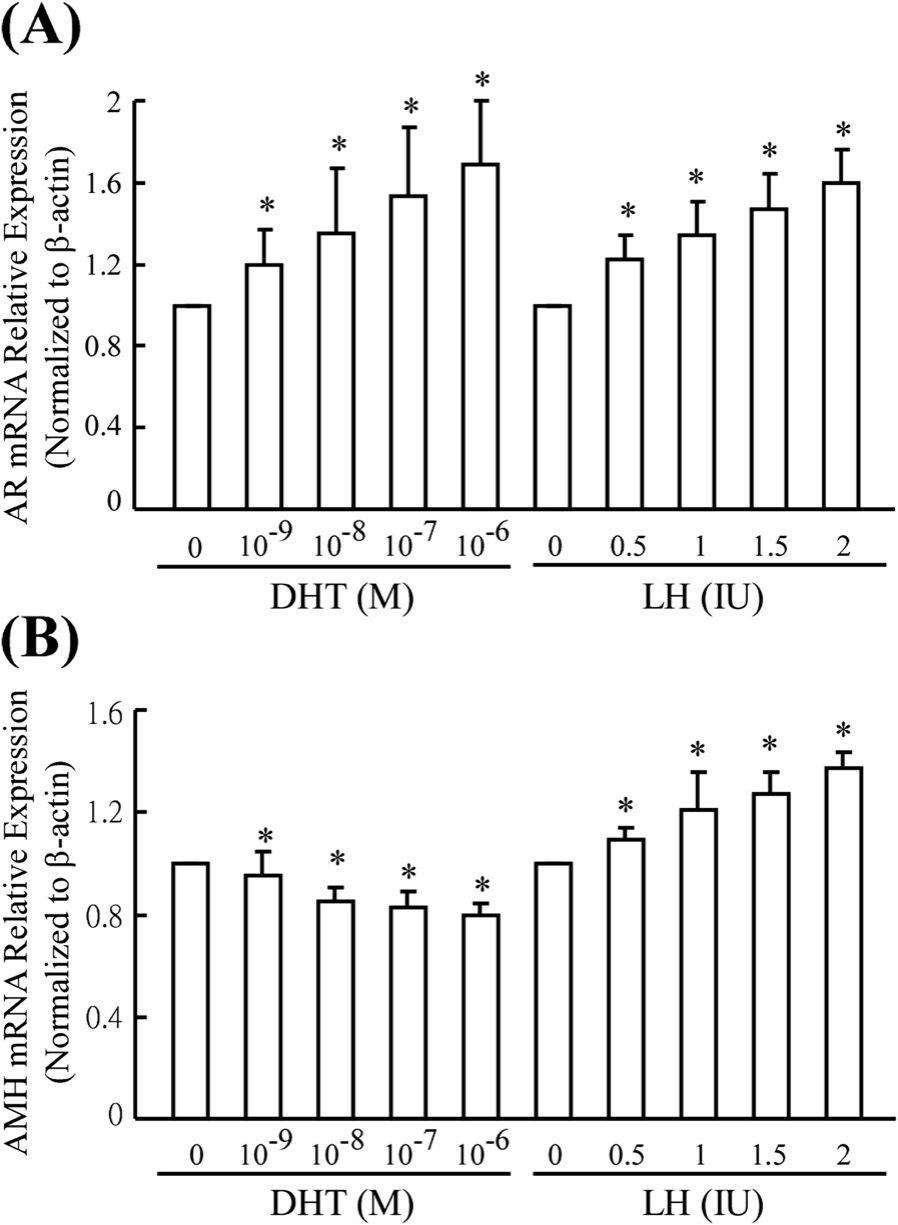
**The effects of rLH and DHT on AR and AMH expression.** (**A**) AR and (**B**) AMH expression levels were measured in HO-23 human granulosa cells under serum-free conditions in the presence of DHT or rLH at the indicated concentrations for 24 hours. Data are expressed as the mean +/- standard deviation (SD) of three different experiments. * *P* < 0.05 vs. ethanol treatment; DHT, dihydrotestosterone; rLH, luteinizing hormone.

To investigate whether androgen or rLH treatment can regulate AR or AMH expression levels *in vitro*, the human granulosa cell line HO23 was treated with increasing doses of DHT (0, 1 x 10^-9^ M, 1 x 10^-8^ M, 1 x 10^-7^ M, or 1 x 10^-6^M) or rLH (0, 0.5 IU/mL, 1 IU/mL, 1.5 IU/mL, or 2 IU/mL). Total RNA was collected 24 hours after treatment, and AR and AMH mRNA levels were measured by quantitative RT-PCR. We observed that increasing the dose of DHT tended to increase AR but decrease AMH expression in HO23 cells. In addition, increases in AR and AMH mRNA expression were observed with increasing doses of rLH (Figure 5 and Additional file 2: Figure S2).

## Discussion

In the present work, we demonstrate that human granulosa-luteal cells supplemented with rLH exhibit increased expression of LHR, AR, SOX9, and AMH. This report is the first study to compare the expression profiles of genes in the androgen receptor and anti-Müllerian hormone in pre-ovulatory granulosa cells from IVF/ICSI patients receiving rLH supplementation for COH. Patients with low expression of AR, AMH, and SOX9 may require a different follow-up schedule and corresponding rLH treatment.

Little is known about the extremely complex process that generates a developmentally competent oocyte. During folliculogenesis and oogenesis, the oocyte is surrounded by granulosa cells. The two cell types communicate bidirectionally through secretion of steroid hormones and paracrine factors. This communication plays a key role in folliculogenesis and is essential for an oocyte to achieve fertilization and undergo embryogenesis [33].

It has been suggested that the expression levels of hormonally-regulated genes in granulosa cells can be used as markers for oocyte quality and hence developmental potential [34,35]. This development is mirrored by the FSH-induced increase in estradiol biosynthesis in granulosa cells after adequate stimulation of theca cells by LH [36]. In 2004, Westergaard et al. found that the follicular fluid concentrations of LH, estradiol and androstenedione were significantly higher and progesterone levels were significantly lower in women treated with gonadotropin plus rLH relative to women treated with recombinant rFSH alone [37]. Smitz et al. also reported that major differences in the serum and follicular fluid endocrine profiles exist after stimulation with gonadotropin and LH, as exogenous LH activity may induce a different endocrine environment [38]. In agreement with these reports [37,38], we found that the addition of rLH was associated with increased estradiol, testosterone, and androstenedione and reduced progesterone in the fluid of the dominant follicle.

AMH is detected in serum from women of reproductive age, and the levels vary.

Slightly with the menstrual cycle, reaching a peak value in the late follicular phase. AMH expression follows a similar pattern in humans compared with mice and rats, suggesting an important role for AMH in folliculogenesis [39]. Wunder et al. [40] reported that serum and follicular fluid AMH levels on the day of oocyte retrieval are correlated with reproductive outcome. From molecular point of view, AMH has been shown to be a downstream target of SOX9. Upregulation of SOX9 can promote the expression of AMH [18]. While increased intrafollicular androgen levels have been associated with significant increases in granulosa cell production of AMH [41], few studies have investigated how reproductive hormones such as LH influence the interaction between the androgen/AR and AMH/SOX9 pathways in granulosa cells.

We demonstrated that luteinized granulosa cells from IVF patients supplemented with rLH display increased LHR, AR, SOX9, and AMH expression and higher testosterone and androstenedione concentrations in the follicular fluid. Androgens are considered detrimental to the late stages of folliculogenesis [42], and hyperandrogenic polycystic ovary syndrome (PCOS) is associated with follicular development arrest and poor oocyte quality [43]. It is possible that LH may stimulate ovarian theca cells to produce androgens that act cooperatively with LH in a paracrine fashion on granulosa cells. Our in vitro model showed that DHT alone can downregulate AMH expression, but increased expression of AR and AMH was observed after rLH treatment. Indeed, many factors [44,45] other than androgens may have affected the expression of AR, AMH and SOX9 under rLH treatment. Increased estradiol or decreased progesterone in the follicular fluid may also influence LH-regulated granulosa cell gene expression. Further studies on interaction between LH and sex steroid hormones will be of interest.

The homogeneity of our study population is an advantage because it reduces the possibility of confounding population substructures or admixtures. However, because our study population was restricted to Taiwanese patients, the results may not be generalizable to other ethnicities. Although our study showed no significant differences in terms of clinical outcomes with or without rLH supplementation (Table 2), the study was underpowered to look at pregnancy rates between the two groups. Our experiments did demonstrate that granulosa-luteal cells supplemented with rLH displayed increased expression of LHR, AR, SOX9, and AMH. As it is difficult to extrapolate the role of rLH in follicular growth and oocyte maturation based on our results, further molecular and cellular functional studies are required to investigate whether rLH therapy has unremarkable benefits or perhaps an undetermined effect on follicular growth and oocyte maturation in women.

## Conclusions

In conclusion, granulosa-luteal cells from women who received rLH supplementation during IVF/ICSI display increased expression of LHR, AR, AMH, and SOX9. AR, SOX9 and AMH were positively correlated and may function in the hormonal milieu affecting late dominant follicles under COH. These markers may be used as a panel of molecular markers during COH.

## Abbreviations

AFC: Antral follicle count; AMH: Anti-Müllerian hormone; AR: Androgen receptor; ARA54: Androgen receptor-associated protein 54; ARA70: Androgen receptor-associated protein 70; ART: Assisted reproductive technology; COH: Controlled ovarian hyperstimulation; Ct: Threshold cycle; DNA: Deoxyribonucleic acid; DHT: Dihydrotestosterone; DMEM: Dulbecco’s minimal essential medium; dNTP: Deoxyribonucleoside triphosphate; FF: Follicular fluid; FSH: Follicle-stimulating hormone; GC: Granulosa cell; GnRHa: Gonadotropin-releasing hormone agonist; HCG: Human chorionic gonadotropin; IUI: Intrauterine insemination; IVF: In vitro fertilization; ICSI: Intracytoplasmic sperm injection; LH: Luteinizing hormone; LHR: Luteinizing hormone receptor; PCOS: Polycystic ovary syndrome; rFSH: Recombinant follicle-stimulating hormone; rLH: Recombinant luteinizing hormone; RNA: Ribonucleic acid; RT-PCR: Reverse transcription polymerase chain reaction; SOX9: Sry-related high-mobility group box protein 9; UNG: Uracil-N-glycosylase

## Competing interests

The authors declare that they have no competing interests.

## Authors’ contributions

KEH and HYK supervised the research. SYC and FJH enrolled the subjects. KCL and HYK conceived and designed the experiments. KCL and HJL carried out the experiments. CYL provided HO23 cells and participated in critical discussion. The manuscript was written by KCL and HYK. All authors read and approved the final manuscript.

## Supplementary Material

Additional file 1: Figure S1 Quantitative analysis of LHR, AR, AMH, SOX9 mRNA levels in granulosa-luteal cells collected at oocyte retrieval. Each bar represents the mean ± SD. Concomitant detection of 18S mRNA in the real-time RT-PCR reaction served as a reference for relative quantification. * *P* < 0.05. Click here for file

Additional file 2: Figure S2 The effects of rLH and DHT on AR and AMH expression. (A) AR and (B) AMH expression levels were measured in HO-23 human granulosa cells under serum-free conditions in the presence of DHT or rLH at the indicated concentrations for 24 hours. Data are expressed as the mean +/- standard deviation (SD) of three different experiments. Concomitant detection of 18S mRNA in the real-time RT-PCR reaction served as a reference for relative quantification * *P* < 0.05 vs. ethanol treatment; DHT, dihydrotestosterone; rLH, luteinizing hormone. Click here for file
