# Supplementary figures and images for: Analysis of androgen receptor and anti-Müllerian hormone pathways in human granulosa cells under luteinizing hormone treatment
Source: Reprod Biol Endocrinol. 2013 Feb 21;11:11. doi: 10.1186/1477-7827-11-11 (PMC3599510; doi:10.1186/1477-7827-11-11)

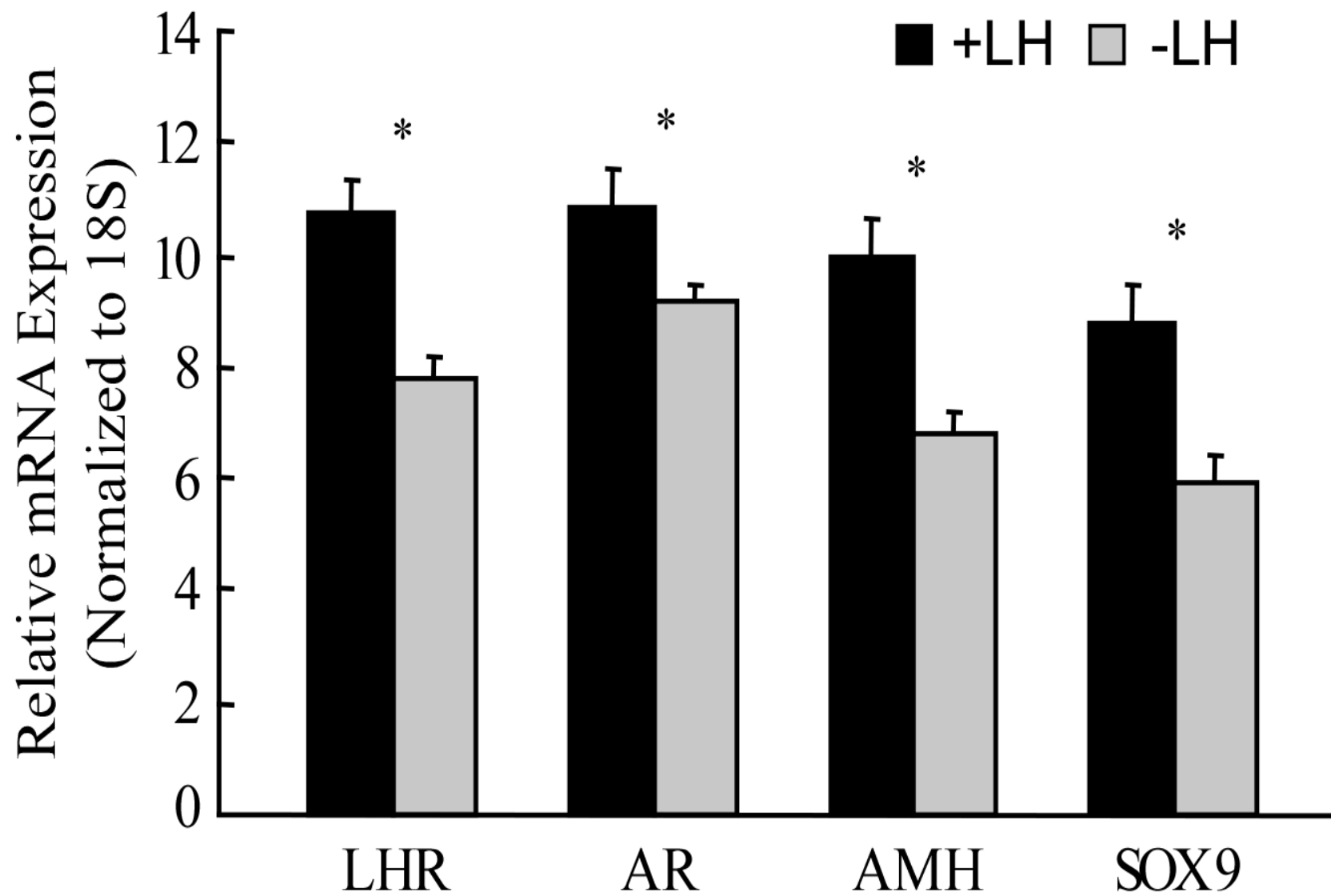

Supplement: Additional file 1: Figure S1 — Quantitative analysis of LHR, AR, AMH, SOX9 mRNA levels in granulosa-luteal cells collected at oocyte retrieval. Each bar represents the mean ± SD. Concomitant detection of 18S mRNA in the real-time RT-PCR reaction served as a reference for relative quantification. * P < 0.05. [file 1477-7827-11-11-S1.pdf]

**(A)**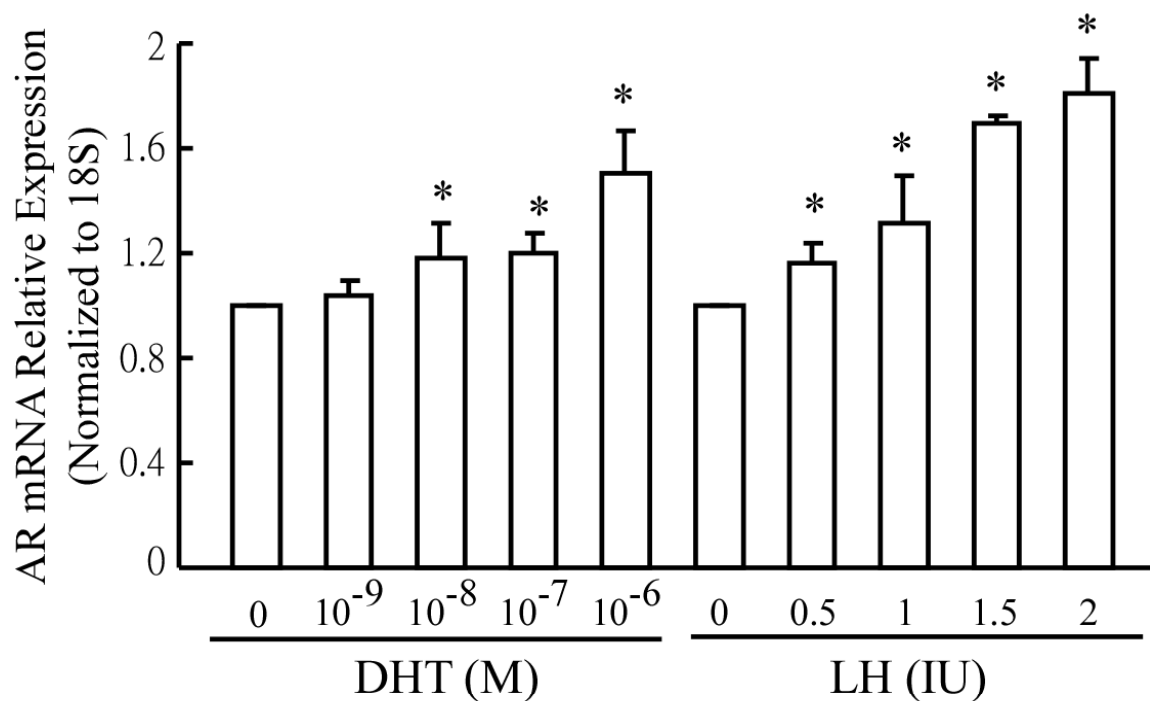**(B)**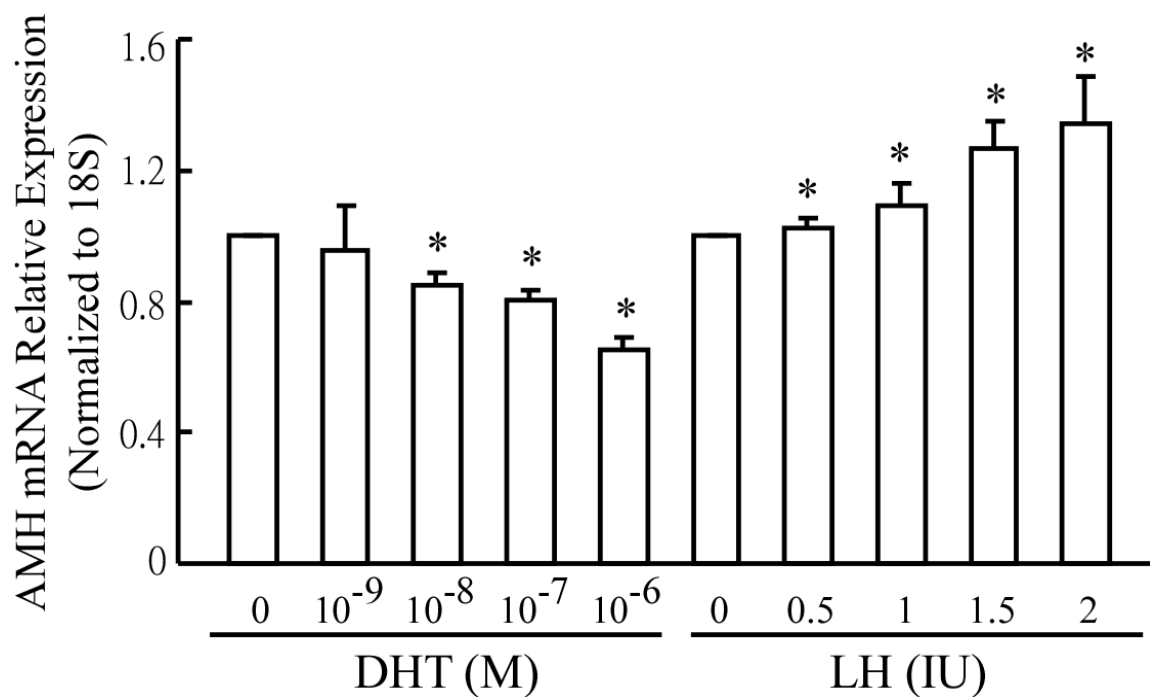

Supplement: Additional file 2: Figure S2 — The effects of rLH and DHT on AR and AMH expression. (A) AR and (B) AMH expression levels were measured in HO-23 human granulosa cells under serum-free conditions in the presence of DHT or rLH at the indicated concentrations for 24 hours. Data are expressed as the mean +/- standard deviation (SD) of three different experiments. Concomitant detection of 18S mRNA in the real-time RT-PCR reaction served as a reference for relative quantification * P < 0.05 vs. ethanol treatment; DHT, dihydrotestosterone; rLH, luteinizing hormone. [file 1477-7827-11-11-S2.pdf]
